# Antisense Oligonucleotide: Basic Concepts and Therapeutic Application in Inflammatory Bowel Disease

**DOI:** 10.3389/fphar.2019.00305

**Published:** 2019-03-29

**Authors:** Davide Di Fusco, Vincenzo Dinallo, Irene Marafini, Michele M. Figliuzzi, Barbara Romano, Giovanni Monteleone

**Affiliations:** ^1^ Department of Systems Medicine, Gastroenterology, University of Tor Vergata, Rome, Italy; ^2^ Department of Pharmacy, School of Medicine and Surgery, University of Naples “Federico II”, Naples, Italy

**Keywords:** inflammatory bowel disease, ulcerative colitis, Crohn’s disease, antisense oligonucleotide, RNA interference

## Abstract

Several molecular technologies aimed at regulating gene expression that have been recently developed as a strategy to combat inflammatory and neoplastic diseases. Among these, antisense technology is a specific, rapid, and potentially high-throughput approach for inhibiting gene expression through recognition of cellular RNAs. Advances in the understanding of the molecular mechanisms that drive tissue damage in different inflammatory diseases, including Crohn’s disease (CD) and ulcerative colitis (UC), the two major inflammatory bowel diseases (IBDs) in humans, have facilitated the identification of novel druggable targets and offered interesting therapeutic perspectives for the treatment of patients. This short review provides a comprehensive understanding of the basic concepts underlying the mechanism of action of the oligonucleotide therapeutics, and summarizes the available pre-clinical and clinical data for oligonucleotide-based therapy in IBD.

## Introduction

The central dogma of molecular biology states that DNA encodes RNA, which is then translated into proteins. In recent years, the use of compounds that are able to bind messenger RNAs (mRNAs) has gained increasing interest as inhibition of protein expression can be helpful for controlling the course of inflammatory and neoplastic diseases. The two major therapeutic approaches in this field are the antisense oligonucleotides (ASOs) that inhibit mRNA translation and the oligonucleotides, which function *via* RNA interference (RNAi) pathway (Chan et al., 2006; Chery, 2016). Synthetic oligonucleotides are negatively charged molecules with different chemical properties based on the technology used for their design. In order to regulate target gene expression, these compounds have to reach disease-associated tissues and cross cell membranes. This is in part facilitated by the manipulation of their chemical structure, which makes oligonucleotides also more powerful and less toxic with a lower chance to have off-target effects and to activate the host immune system (Sharma and Watts, 2015).

In the last decades, the advent of new techniques of molecular and cellular biology has advanced our understanding of the factors/mechanisms that promote tissue damage in several chronic inflammatory diseases, including Crohn’s disease (CD) and ulcerative colitis (UC), the two major inflammatory bowel diseases (IBDs) in humans (Neurath, 2017). This has contributed in identifying novel druggable targets, thus offering interesting therapeutic perspectives for the treatment of these patients. We here shortly review the basic concepts underlying the mechanism of action of the ASOs and summarize the available data for ASO-based therapy in IBD.

## Antisense Oligonucleotide Strategy and Molecule Design

**Figure 1:**
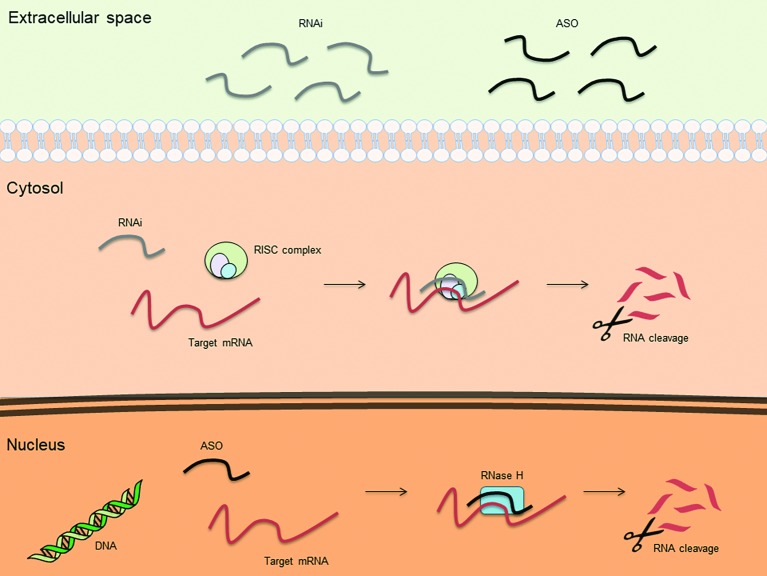
Basic mechanisms of action for therapeutic antisense oligonucleotides (ASOs) and RNA interference (RNAi).

An antisense oligonucleotide (ASO) is a single-stranded deoxyribonucleotide, which is complementary to the mRNA target. The goal of the antisense approach is the downregulation of a molecular target, usually achieved by induction of RNase H endonuclease activity that cleaves the RNA-DNA heteroduplex with a significant reduction of the target gene translation (Figure 1). Other ASO-driven mechanisms include inhibition of 5′ cap formation, alteration of splicing process (splice-switching), and steric hindrance of ribosomal activity (Chan et al., 2006; Bennett et al., 2017; Crooke, 2017).

The recent developments in the human genome sequencing, the possibility of a rational design of oligonucleotides and the theoretical simplicity, and relatively cheap costs of these compounds led to their use as either therapeutic agents or tools for assessing gene function. Although, the researchers usually select the ASO candidate by testing the activity of few oligonucleotides that specifically regulate the target gene expression, it would be recommendable to identify the ideal ASO through an accurate evaluation of a panel of putative oligomers (Tu et al., 1998; Stein, 2001). It is crucial that the ASOs do not bind, even partially, to a nontarget mRNA. In this context, it is noteworthy that 6–7 base pairs between the ASO and nontarget mRNA are sufficient to initiate RNase activity, leading to cleavage of the wrong target. The secondary and tertiary structure of the RNA must be taken into account to minimize the possibility that the selected sequence is inaccessible to binding (Ho et al., 1998; Vickers et al., 2000; Andronescu et al., 2005). To this end, the use of software with a robust RNA folding program (e.g., Sfold or mfold) can help select the optimal candidate (Zuker, 2003; Ding et al., 2004). Generally, the length of an ASO is approximately 20 nucleotides and the ASO is selected to target either the methionine (AUG) initiation codon (to block translation) or splice sites (to block splicing) (Chan et al., 2006; Chery, 2016). The effective knockdown of the target is usually demonstrated at the protein level, but analysis of RNA expression should be made in order to exclude that the target protein is down-regulated through a non-sequence specific mechanism. To maximize sequence specificity, ASOs should not be designed into polymorphic/mutated regions of the genome and selection should exclude oligonucleotides targeting four contiguous guanosine residues in order to avoid generation of tetraplexes via Hoogsteen base-pair formation (Sen and Gilbert, 1992; Benimetskaya et al., 1997; Crooke, 2004).

Since in their “naïve” form, ASOs could be rapidly digested, thus limiting their bioavailability (Eder et al., 1991), most of ASOs are phosphorothioated (Eckstein, 2014). This modification facilitates binding of ASOs to plasma proteins, thereby reducing their renal loss and improving uptake to several organs (e.g. liver, bone marrow, and lymph nodes). The chemical modification influences neither RNase H activity nor ASO solubility, thus allowing administration by different routes (e.g. subcutaneous, intravenous, topical, oral, or rectal). However, phosphorothioate oligonucleotides containing one or more CpG motifs can bind the Toll-like receptor (TLR) 9 and trigger innate immune responses. This issue can be overcome by either selecting oligonucleotides containing no CG or replacing the C with 5methylC, which does not stimulate the immune system (Stein, 2001).

Increased ASO binding affinity and biostability have also been obtained using oligonucleotides with ribose modifications [i.e. substitution of the hydrogen at the 2-position by an O-alkyl group and locked nucleic acid technology (LNA)] that reduce conformational plasticity (Wahlestedt et al., 2000; Prakash, 2011; Hagedorn et al., 2018). However, LNA can accumulate in the liver and promote hepatotoxicity, mainly due to an off-target RNase H dependent RNA degradation (Burel et al., 2016).

ASOs have been already used in various human pathologies. For instance, in 1998, FDA approved the use of fomivirsen, a compound that inhibits the translation of the mRNA encoding for the major immediate early region proteins of cytomegalovirus, for the treatment of cytomegalovirus-induced retinitis (Jabs and Griffiths, 2002). In 2013, FDA approved the use of mipomersen, a compound targeting apolipoprotein B100, for the treatment of familial hypercholesterolemia (Duell et al., 2016), while later on, eteplirsen was introduced to treat Duchenne muscular dystrophy, and nusinersen was approved for spinal muscular atrophy treatment (Khorkova and Wahlestedt, 2017; Goyal and Narayanaswami, 2018). Eteplirsen binds to the disease-related-exon 51 of dystrophin RNA and allows the splicing of exon 52 to exon 51, thus generating a shortened but partly functional protein (exon skipping strategy) (Khorkova and Wahlestedt, 2017). Differently, nusinersen uses an exon switching strategy to increase the amount of functional full-length survival motor neuron-2 protein. After hybridization to its target, this oligonucleotide forces the inclusion of exon 7 into the mRNA and prevents the generation of short-lived/non-functional proteins (Goyal and Narayanaswami, 2018). Clinical trials employing ASOs in amyotrophic lateral sclerosis and familial amyloid polyneuropathy are also ongoing (Goyal and Narayanaswami, 2018).

## Antisense Oligonucleotide-Based Therapies for Inflammatory Bowel Disease

### Alicaforsen: Intercellular Adhesion Molecule-1 Antisense Oligonucleotide

IBD are chronic, immune-mediated diseases of the gastro-intestinal tract, which are characterized by tissue damage and development of local and extra-intestinal lesions (Abraham and Cho, 2009; Neurath, 2017). One of the mechanisms sustaining the inflammatory process in IBD is the recruitment of immune cells from the peripheral blood to the intestine. Once activated in secondary lymphoid organs, such as Peyer’s patches and isolated follicles, leukocytes enter the circulation, and through a process named gut homing, eventually go back to the intestinal wall. This process is triggered mainly by chemoattractants produced within the inflamed tissue and favored by interaction between integrins expressed on leukocyte surface and proteins expressed on endothelial cells, such members of immunoglobulin superfamily [i.e. intercellular adhesion molecule (ICAM)-1, ICAM-2, and vascular cell adhesion molecule (VCAM)-1] (Hart et al., 2010).

In inflamed gut of CD patients and UC patients, there is an enhanced expression of ICAM-1, a transmembrane glycoprotein constitutively expressed on the surface of intestinal epithelial cells and vascular endothelial cells (Vainer and Nielsen, 2000). Knockdown of ICAM-1 with specific ASO in mouse models of colitis reduced leukocyte trafficking to the gut and attenuated mucosal inflammation (Bennett et al., 1997). In a proof of concept study, Alicaforsen (ISIS 2302), a 20 base-long phosphorothioate ASO inhibiting ICAM1 production, was intravenously administered to 20 active CD patients for 26 days. The drug was well tolerated and superior to placebo in inducing clinical remission (Yacyshyn et al., 1998). However, steroid-dependent or resistant CD patients treated with intravenous or subcutaneous alicaforsen in two subsequent clinical trials showed no clinical benefit (Schreiber et al., 2001; Yacyshyn et al., 2002). Similar negative results were also obtained in two subsequent placebo-controlled phase III trials, in which alicaforsen was given to moderate-to-severe active CD patients (Yacyshyn et al., 2007). Therefore, the therapeutic development of alicaforsen in CD was discontinued.

An alicaforsen-containing enema formulation was developed for patients with UC or patients with pouchitis, an inflammatory condition of the ileal pouch reservoir, which can develop in UC patients undergoing colectomy and ileal pouch-anal anastomosis. In mild to moderate left-sided UC patients, alicaforsen enema had no significant effect on the course of the disease (Miner et al., 2006; Van Deventer et al., 2006). Afterwards, a retrospective analysis evaluating the efficacy of alicaforsen enema (240 mg/day for 6 weeks) showed clinical benefits in patients with left-sided and distal UC and in patients with chronic pouchitis (Greuter et al., 2018). However, treatment was not sufficient to stably control the inflammation as more than 2/3 of the patients relapsed within 16 weeks (Greuter et al., 2016). A phase III, multicenter, double-blind, placebo-controlled trial (NCT02525523) assessing the safety and efficacy of topical alicaforsen enema (240 mg/day for 6 weeks) has been recently completed in subjects with antibiotic refractory pouchitis but results are not yet available.

### NF-κB Antisense Oligonucleotide

NF-κB is a transcription factor composed of two proteins (p50 and p65) regulating the expression of many inflammatory and anti-inflammatory genes (Rogler et al., 1998; Schreiber et al., 1998). It was shown that either intravenous or intra-rectal ASO targeting the p65 subunit of NF-κB inhibited production of inflammatory cytokines and signs of colitis induced in mice by trinitrobenzene sulfonic acid (TNBS) or IL-10 deficiency (Neurath et al., 1996). Consistently, the specific p65 ASO reduced production of inflammatory cytokines in macrophages and endothelial cells isolated from the gut of CD patients (Neurath et al., 1998). These data were in line with the demonstration that downregulation of NF-κBp65 with a specific ASO attenuated dextran sodium sulfate (DSS)-induced colitis (Murano et al., 2000) and intestinal fibrogenic processes in mice (Lawrance et al., 2003). Despite these encouraging data in IBD-like murine models, no data are currently available on the use of NF-κB ASO in IBD.

### Smad7 Antisense Oligonucleotide

IBD is believed to be triggered by complex interactions among host genetic susceptibility and many environmental factors, which lead to a sustained activation of inflammatory pathways and defects in counter-regulatory mechanisms in the gut (Gorelik and Flavell, 2002; MacDonald et al., 2011). In intestinal immunity, transforming growth factor (TGF)-β1, a pleiotropic cytokine produced by many cell types suppresses inflammatory responses to luminal antigens, thus contributing to immune tolerance induction. The anti-inflammatory mechanism of TGF-β1 relies mainly on the intracellular phosphorylation and subsequent activation of TGF-β1 receptor-associated Smad2/3 proteins (Heldin et al., 1997; Shi and Massague, 2003). In IBD patients, phosphorylated-Smad2/3 expression is reduced thus underlying the inability of TGF-β1 to adequately control inflammatory signals (Babyatsky et al., 1996). Such a defect has been associated with increased levels of Smad7, a cytosolic protein that inhibits TGF-β1/Smad-associated pathway (Monteleone et al., 2001; Sedda et al., 2015). Responsiveness of IBD mucosal cells to TGF-β1 is restored by downregulation of Smad7 with a specific ASO (Monteleone et al., 2001). Oral administration of Smad7 ASO to mice with TNBS and oxazolone-induced colitis restores TGF-β1-associated Smad signaling and mitigates intestinal inflammation (Boirivant et al., 2006).

Later on, a pharmaceutical compound, which contains a Smad7 ASO targeting the RNA encoding by the 107–128 DNA region, was developed for CD therapy. The drug, named mongersen (previously called GED-0301), (Monteleone et al., 2012; Laudisi et al., 2016), was formulated in order to maximize the active compound release into the lumen of the terminal ileum and right colon, the intestinal regions mainly involved in CD. A phase I clinical, open-label, dose-escalating study in patients with active, steroid-dependent/resistant CD showed that mongersen was safe and well-tolerated and treatment was associated with a clear clinical benefit (Monteleone et al., 2012). Although TGF-β1 is known to be pro-fibrogenic (Leask and Abraham, 2004; Vallance et al., 2005), no patient recruited into the trial developed strictures (Zorzi et al., 2012). This later result was consistent with data generated in mice with TNBS-mediated colitis-driven intestinal fibrosis, in which knockdown of Smad7 with the specific ASO reduced intestinal inflammation and fibrosis (Izzo et al., 2018). A double blind, placebo controlled, phase II trial was conducted in 166 active, steroid-dependent/resistant CD patients (Monteleone et al., 2015). Patients were allocated to receive one of three doses of mongersen (10, 40, or 160 mg per day) or placebo daily for 2 weeks. Patients receiving the 40 and 160 mg of mongersen reached significant higher rates of remission (55 and 65%, respectively) than those treated with 10 mg or placebo (12 and 10%, respectively). At the end of follow-up, the percentage of patients who had a steroid-free remission was significantly greater in the 160-mg group than in the placebo group. The study confirmed the safety profile of the drug (Monteleone et al., 2015). These data were confirmed by a subsequent multicenter, randomized study, which evaluated the effect of mongersen on endoscopic outcomes (Feagan et al., 2018). Sixty-three active CD patients were randomized (1:1:1) to 4, 8, or 12 weeks of oral mongersen (160 mg daily). Endoscopic improvement was observed in 37% of participants. All three mongersen regimens induced rapid, clinically meaningful decreases in Crohn’s disease activity index scores. Moreover, reductions in high-sensitivity C-reactive protein levels and fecal calprotectin were observed in patients with increased values at baseline (Feagan et al., 2018). A phase III clinical trial has been recently suspended due to an interim analysis documenting the lack of efficacy of mongersen. The reasons for this unexpected result are still to be clarified.

## RNA Interference Strategy and its Therapeutic Application

Another strategy to inhibit the expression of mRNA is represented by RNAi. RNAi is mediated by many endogenous RNAs [e.g. piwi-interacting RNA (piRNA), microRNA (miRNA), and small interfering RNA (siRNA) (Li and Rana, 2012)]. Once incorporated into the RNA-induced silencing complex (RISC), these RNAs cause translational repression/degradation of the targeted mRNA through the partial or complete base paring of the guide strand (Figure 1). This result can be obtained by using single stranded RNAs (ssRNAs), which can be directly incorporated into RISC, or double stranded RNAs (dsRNAs), which require cleavage by the cytoplasmic endoribonuclease Dicer prior to be incorporated into RISC (Li and Rana, 2012). Due to their chemical characteristics, synthetic silencing RNAs do not efficiently enter into cells and are highly susceptible to nuclease degradation. To overcome these limitations, silencing RNAs can be complexed with nanoparticles, typically as polymer- or lipid-based formulations (Tatiparti et al., 2017). It is, however, noteworthy that nanoparticles can increase the toxicity of the compound or alter pharmacokinetics and biodistribution of the silencing RNAs. Patisiran is the first RNAi-based drug approved by FDA for the treatment of polyneuropathy caused by hereditary transthyretin-mediated amyloidosis (hATTR). It consists of a dsRNA encapsulated in a nanoparticle that allows the active molecule to reach the liver, where it specifically inhibits the hepatic synthesis of transthyretin (Adams et al., 2018). It remains to be clarified whether this strategy can be effective in organs other than liver, where delivery could be more difficult.

Another strategy is to conjugate the silencing RNAs with ligands of the target molecule. An example is the addition of N-acetylgalactosamine (GalNAc) to the RNA, thus enhancing asialoglycoprotein receptor (ASGR)-mediated uptake into liver hepatocytes (Nair et al., 2014). The major limitation of this strategy could be the rate of receptor recycling. GalNAc delivery is actually involved in several clinical and pre-clinical studies with exciting results (Tanowitz et al., 2017).

## Small Interfering RNA-Based Therapies for Inflammatory Bowel Disease

### STNM01: Small Interfering RNA Targeting Carbohydrate Sulfotransferase 15

The late stage of inflammation in IBD is characterized by the fibrotic process, which derives from an altered balance between matrix deposition and degradation (Rieder et al., 2017). Carbohydrate sulfotransferase 15 (CHST15) is a sulfotransferase responsible for biosynthesis of chondroitin sulfate E-type (CS-E), which binds to pro-inflammatory and pro-fibrotic mediators, adhesion molecules, receptor for advanced glycation end-product (RAGE), and pathogenic microorganisms, all of them involved in fibrogenesis. CHST15 is increased in the colon of active CD patients (Belmiro et al., 2005; Suzuki et al., 2016, 2017). STNM01, a synthetic, double-stranded RNA oligonucleotide directed against CHST15, ameliorated acute and chronic DSS induced-colitis and reduced colonic deposition of collagen in mice (Suzuki et al., 2016). A phase 1, randomized, double blind, placebo-controlled, clinical trial evaluated the safety of STNM01 in patients with CD (Suzuki et al., 2017). Eighteen CD patients with mucosal lesions refractory to conventional therapies received a single-dose, endoscopic, submucosal injection of 2.5, 25, or 250 nM STNM01 (three patients per group) or placebo (nine patients). The drug was well tolerated, CHST15 expression was reduced 1 month after the injection, and the drug attenuated intestinal inflammation and fibrosis.

## Conclusion

The rationale for the use of antisense-based therapies in IBD is supported by the benefit seen in preclinical models and initial clinical studies, together with the safety profiles of the compounds. Unfortunately, however, large clinical trials have not confirmed the promising results obtained with ASOs in preclinical models. Although, it is unclear why these treatments failed in patients, it is conceivable that some factors either related to the target or route of administration may have contributed to these negative results. For example, the negative results of alicaforsen can, in part, rely on the fact that ICAM-1 is just one of the various molecules involved in leukocytes trafficking, and therefore, even in the absence of ICAM-1, other integrins could promote recruitment of activated leukocytes in the gut. Another possibility is that systemic administration of ASO could be not ideal for allowing optimal concentration of the drug within the gut tissue, where there is the main expression of the target. This hypothesis is supported by the demonstration that rectal administration of alicaforsen is of benefit in patients with distal UC and in patients with pouchitis. While STNM01 is the only siRNA currently tested in IBD, there is sufficient evidence to believe that RNAi technology can represent a new and valid approach to regulate the expression of disease-related genes. Some issues in the design and development of these compounds, such as correct identification of target mRNAs, stability, and delivery to the site of interest remain to be solved.

## Data Availability

All datasets generated for this study are included in the manuscript.

## Author Contributions

DF and VD wrote the paper. MF, IM and BR contributed to supervise parts of the paper. GM designed and drafted the paper.

### Conflict of Interest Statement

GM has filed a patent related to the treatment of IBD with Smad7 antisense oligonucleotides.The remaining authors declare that the research was conducted in the absence of any commercial or financial relationships that could be construed as a potential conflict of interest.
